# Replication of SNP associations with keratoconus in a Czech cohort

**DOI:** 10.1371/journal.pone.0172365

**Published:** 2017-02-16

**Authors:** Petra Liskova, Lubica Dudakova, Anna Krepelova, Jiri Klema, Pirro G. Hysi

**Affiliations:** 1 Institute of Inherited Metabolic Disorders, First Faculty of Medicine, Charles University and General University Hospital in Prague, Prague, Czech Republic; 2 Department of Ophthalmology, First Faculty of Medicine, Charles University and General University Hospital in Prague, Prague, Czech Republic; 3 Department of Biology and Medical Genetics, Second Faculty of Medicine, Charles University and University Hospital Motol, Prague, Czech Republic; 4 Department of Computer Science, Czech Technical University in Prague, Prague, Czech Republic; 5 KCL Department of Ophthalmology, King’s College London and Great Ormond Street Institute of Child Health, University College London, London, United Kingdom; Universita degli Studi di Roma Tor Vergata, ITALY

## Abstract

**Introduction:**

Keratoconus is a relatively frequent disease leading to severe visual impairment. Existing therapies are imperfect and clinical management may benefit from improved understanding of mechanisms leading to this disease. We aim to investigate the replication of 11 single nucleotide polymorphisms (SNPs) with keratoconus.

**Methods:**

SNPs from loci previously found in association with keratoconus were genotyped in 165 keratoconus cases of Caucasian Czech origin (108 males and 57 females) and 193 population and gender-matched controls. They included rs1536482 (*COL5A1)*, rs4839200 (*KCND3)*, rs757219 and rs214884 (*IMMP2L)*, rs1328083 and rs1328089 (*DAOA)*, rs2721051 (*FOXO1)*, rs4894535 (*FNDC3B)*, rs4954218 (*MAP3K19*, *RAB3GAP1)*, rs9938149 (*ZNF469)* and rs1324183 (*MPDZ)*. A case-control association analysis was assessed using Fisher’s exact tests.

**Results:**

The strongest association was found for rs1324183 (allelic test OR = 1.58; 95% CI, 1.10–2.24, p = 0.01). Statistically significant values were also obtained for rs2721051 (allelic test OR = 1.72; 95% CI, 1.07–2.77, p = 0.025) and rs4954218 (allelic test OR = 1.53; 95% CI, 1.01–2.34; p = 0.047) which showed an opposite effect direction compared to previously reported one.

**Conclusion:**

Independent replication of association between two SNPs and keratoconus supports the association of these loci with the risks for the disease development, while the effect of rs4954218 warrants further investigation. Understanding the role of the genetic factors involved in keratoconus etiopathogenesis may facilitate development of novel therapies and an early detection.

## Introduction

Keratoconus (KC) is a progressive eye disoder characterized by non-inflammatory central or paracentral corneal thinning and steepening leading to irregular astigmatism that causes a decrease in visual acuity. In patients with advanced disease there is also significant corneal scarring. The disease is almost always bilateral although there may be marked asymetry. Onset occurs typically in early adolescence and progression stops before the age of 40 years. [1]. KC is a relatively common disorder, affecting approximately 1 in 2,000 people [2]. Although many cases appear sporadic, familial aggregations and twin studies suggest a strong genetic component in the KC development [3–5].

Specific genetic factors contributing to the risk of KC remain largely unknown. Two relatively underpowered genome-wide association studies (GWAS) using single nucleotide polymorphisms (SNPs) markers have been performed to date and identified several variants reportedly influencing susceptibility to KC [6,7]. To date, only the association of KC with rs2956540 in *LOX* and rs3735520 in the promoter of *HGF* has been independently replicated in individuals of European descent [8]. The *LOX* gene is also located beneath the linkage peak reported in a genome-wide linkage analysis in KC sib pair families genotyped with microsatellite markers [9].

As corneal thinning is a known genetically correlated endophenotype for KC [10], quantitative explorations of the genetic architecture of KC has attempted through associations with KC of central corneal thickness (CCT) genetic risk loci [10,11]. Li et al. (2012) [7] and Lu et al. (2013) [10] reported five loci (six independent SNPs) significantly associated with keratoconus. Of these, effects of two SNPs rs1324183 upstream of *MPDZ* and rs9938149 upstream of *ZNF469* genes have been confirmed in relation to KC risk in an independent cohort of patients of European origin [12].

The relatively low prevalence of KC has constrained both the power of initial discovery cohorts as well as the possibilities of independent replication; indeed most variants identified did not reach in discovery stages commonly considered genome–wide significance thresholds. Yet independent replications are an integral part of the genetic association studies as, among other reasons, they contribute to the increasing credibility of GWAS, improve the accuracy of the effect size calculation and help in the elimination process of false positive findings. Given the general lack of independent replication for KC loci, additional evidence of genetic association, whether positive or negative, will improve our understanding of genetic factors and mechanisms truly influencing KC in the general population. In the current study we genotyped and analyzed in a Czech case-control panel 11 SNPs previously reported to be associated with the risk of KC development in cases of European descent.

## Materials and methods

The study was approved by the Ethics Committee of the General Teaching Hospital and Charles University, Prague. Sample collection and experiments were conducted according to the principles set out in the Declaration of Helsinki. Written, informed consent was obtained from all individuals.

A total of 165 unrelated KC cases, 57 females and 108 males, participated in the study. All cases were reported for care at the Department of Ophthalmology in Prague between 2011 and 2013, and exhibited KC pattern on front sagittal curvature maps, together with localized corneal thinning in at least one eye, or had and an advanced disease with stromal scarring, Fleischer ring and Vogt striae [1]. Only patients with topographic KC index grade 1 or higher, as calculated by Pentacam build-in KC detection software (Oculus Optikgeräte GmbH, Wetzlar, Germany), were included [13]. Bilaterally grafted patients for KC were also considered as cases. 193 unrelated Czech Caucasian individuals (79 females, 114 males) self-reporting no ocular disorders impairing vision were used as controls.

DNA was extracted from venous blood using Gentra Puregene Blood Kit (QIAGEN, Dusseldorf, Germany) and AutoGenFlex STAR system (AutoGen, Holliston, MA, USA), and from saliva (Oragene OG-300, DNA Genotek, Canada). Kompetitive Allele Specific PCR (KASP) assays (LCG Genomics, UK) were used to genotype 11 SNPs (Table 1). Five of the tested loci near the *ZNF469* (rs9938149), *FOXO1* (rs2721051), *COL5A1* (rs1536482), *FNDC3B* (rs4894535) and *MPDZ* (rs1324183) genomic sequences, were primarily associated with CCT [10]. The *IMMP2L* (rs757219, rs214884), *KCND3* (rs4839200), *DAOA* (rs1328089, rs1328083) and *RAB3GAP1/MAP3K19* (rs4954218) loci were identified through KC in GWAS [7,14]. Details of previous associations for the 11 SNPs tested in the current study and their results are summarized in S1 and S2 Tables. KASP genotyping is based on competitive allele-specific PCR. The two allele-specific forward primers each contain a unique tail sequence labelled with different fluorescent dyes. Bi-allelic discrimination is achieved through their competitive binding. If the genotype at a given SNP is homozygous, only one of the two possible fluorescent signals is generated, heterozygous genotype leads to mixed fluorescent signal [15].

**Table 1:** Analyzed SNPs and results of their association testing with keratoconus in a Czech case-control panel.

					Allelic Test					Effect direction in comparison with discovery studies	Dominant model			Recessive model		
SNP	Chr. position	Original phenotype	Nearest gene	Minor /major allele	MAF Cases	MAF Controls	OR	Exact 95% CI	Exact p-value		OR	Exac 95% CI	Exact p-value	OR	Exact 95% CI	Exac p-value
rs4839200	1:112021228	KC	31.7 kb upstream of *KCND3*	A/G	0.127	0.135	0.94	0.60–1.46	0.82	Opposite	0.82	0.49–1.35	0.46	2.38	0.55–11.17	0.31
rs4954218	2:135045855	KC	*MAP3K19;* intron 1 Upstream of *RAB3GAP1*	G/T	0.182	0.127	1.53	1.01–2.34	**0.047**	Opposite	1.47	0.90–2.39	0.12	2.72	0.85–9.69	0.098
rs757219	7:111082426	KC	*IMMP2L;* intron 4	G/A	0.130	0.166	0.75	0.49–1.15	0.21	Opposite	0.69	0.43–1.11	0.12	1.17	0.28–4.98	1.00
rs214884	7:111106552	KC	*IMMP2L;* intron 4	C/T	0.094	0.111	0.83	0.51–1.35	0.46	Opposite	0.85	0.50–1.45	0.59	0.39	0.01–3.55	0.63
rs1328083	13:105800628	KC	309.6 kb downstream of *DAOA*	G/T	0.161	0.145	1.13	0.74–1.71	0.60	Consistent	1.08	0.66–1.75	0.81	1.66	0.49–5.48	0.56
rs1328089	13:105815706	KC	324.7 kb downstream of *DAOA*	C/T	0.252	0.233	1.11	0.78–1.57	0.60	Consistent	1.11	0.72–1.73	0.67	1.18	0.50–2.78	0.83
rs4894535	3:172277815	CCT/KC	*FNDC3B;* intron 7	T/C	0.173	0.168	1.03	0.69–1.53	0.92	Consistent	1.03	0.64–1.63	0.91	1.17	0.21–6.56	1.00
rs1536482	9:134548682	CCT/KC	93.1 kb upstream of *COL5A1*	A/G	0.358	0.293	1.34	0.98–1.86	0.066	Consistent	1.31	0.86–2.04	0.20	1.92	0.94–3.93	0.084
rs1324183	9:13557492	CCT/KC	277.8 kb upstream of *MPDZ*	A/C	0.279	0.197	1.58	1.10–2.24	**0.01**	Consistent	1.76	1.13–2.72	**0.0096**	1.56	0.66–3.71	0.39
rs2721051	13:40536747	CCT/KC	18.9 kb downstream of *FOXO1*	T/C	0.142	0.088	1.72	1.07–2.77	**0.025**	Consistent	1.65	0.96–2.82	0.068	5.97	0.81–140.46	0.099
rs9938149	16:88298034	CCT/KC	129.4 kb upstream of *ZNF469*	C/A	0.336	0.378	0.83	0.61–1.14	0.27	Consistent	0.79	0.51–1.22	0.29	0.82	0.44–1.49	0.56

Chromosomal position corresponds to the human assembly Dec. 2013 (GRCh38). The alleles of the genomic sequence refer to the positive strand and ancestral alleles are derived from dbSNP. The effect sizes are reported with reference to the minor allele. Effect direction is a comparison with the original discovery study reporting on the SNP association. The p-values for differences between patients and controls were calculated using Fisher’s exact test and were rounded up to the second relevant decimal.

**MAF** = minor allele frequency; **OR** = odds ratio for the minor allele; **CI** = confidence interval; Effect direction is given with reference to the directionality of effect in the first published association report; NA = not available; ***KCND3*** = potassium channel, voltage gated Shal related subfamily D, member 3; ***MAP3K19*** = mitogen-activated protein kinase kinase kinase 19, NM_001018044.2; ***FNDC3B*** = fibronectin type III domain containing 3B, NM_022763.3; ***IMMP2L*** = IMP2 inner mitochondrial membrane peptidase-like (*S*. *cerevisiae*), NM_032549.3; ***COL5A1*** = collagen, type V, alpha 1; ***MPDZ*** = multiple PDZ domain protein; ***DAOA*** = D-amino acid oxidase activator; ***FOXO1*** = forkhead box O1; ***ZNF469*** = zinc finger protein 469

Statistically significant p-values <0.05 are indicated in bold.

To test for association, Fisher’s exact test odds ratios (ORs) and p-values were calculated for each SNP, under an allelic test model which assumes co-dominance, using the ‘exact2x2’ package in R (https://cran.r-project.org). Secondary analyses of association under assumptions of dominant and recessive models were calculated for each SNP. An α<0.05 was considered as the threshold for statistical significance, on the grounds these variants were pre-selected because of strong prior probabilities of association. Allele frequencies in the control group were compared to European panels in the HapMap project (http://hapmap.ncbi.nlm.nih.gov/).

Conventional Sanger sequencing was performed when samples had DNA concentration not reaching requirements for KASP assays and in cases of genotype calling failure. In total, 1.57% genotypes (62 out of 3960) were established using this method. In addition, for internal data quality, all 11 SNPs were Sanger-sequenced in 5 randomly selected samples (primers sequences are listed in S3 Table).

## Results

The mean age of the cases was 37.18 years (standard deviation 13.26, range 15–69 years) whereas the controls were in average 39.46 years old at recruitment (standard deviation 13.68, range 20–81 years). The sex ratio was also similar between cases and controls (65% male cases compared to 60% male controls).

All SNPs in controls had allele frequencies similar to publicly available European reference panels and were in Hardy-Weinberg equilibrium (p>0.05).

In total, 3 out of the 11 SNPs, crossed the pre-established threshold for association significance with KC under the assumptions of our primary model for additive inheritance (Table 1); the rs1324183, upstream of *MPDZ* (OR = 1.58; 95% CI, 1.10–2.24, p = 0.01), rs2721051 downstream of *FOXO1* (OR = 1.72; 95% CI, 1.07–2.77, p = 0.025) and rs4954218 (OR = 1.53; 95% CI, 1.01–2.34; p = 0.047) in an intron of *MAP3K19* and upstream of *RAB3GAP1* gene. A fourth SNP (rs1536482, upstream *COL5A1*) had a suggestive, albeit not formally significant association with KC case-control status (p = 0.066). Evidence for association did not improve when other inheritance models were assumed, except for rs1324183 (*MPDZ* locus) whose association significance slightly increased under a dominant model for allele A (p = 0.0096).

## Discussion

Out of 11 SNPs previously reported to influence the risk of KC in predominantly Caucasian cohorts (S1 and S2 Tables) [7,10,14], we have observed that in the Czech case-control panel rs1324183 (*MPDZ* locus) confers the strongest and most significant risk compared to all other SNPs included in our genotyping panel. Other variants that significantly contributed to KC genetic risk in our samples were rs2721051 (*FOXO1* locus) and rs4954218 (*MAP3K19*/*RAB3GAP1* locus). For all variants, our results appear to support an additive genetic model since secondary analyses under assumptions of different modes of inheritance (dominant or recessive) failed to substantially improve the strength of association.

All studies performed on populations of European descent (S1 and S2 Tables), including the Czech one, demonstrated that the minor allele of rs1324183 (*MPZD*) serves as a risk factor.

Surprisingly the effect sizes of four SNPs out of six initially associated in the original KC GWAS went in the opposite way in our samples, whereas for those initially associated with CCT (and KC); the effect size consistently went in the same direction.

Contrary to the previous studies testing individuals of European descent, where minor allele of rs4954218 (*MAP3K19*/*RAB3GAP1*) showed a protective effect, in the Czech cohort it conferred a statistically significant (p<0.05) risk for KC [7,14]. There are several possible explanations for this inconsistent direction of effect. First, it may be a case of type I or type II error in either study, especially in consideration of the modestly powered samples involved. Second, this may be another instance of the so-called “flip-flop phenomenon” [16] also seen in genetic literature for other ophthalmic diseases [17] presumably due to variations in linkage disequilibrium with the causative alleles in different populations. Future studies, using even larger number and potentially more ethnically diverse KC samples may be needed to clarify definitely the role of the rs4954218 (*MAP3K19*/*RAB3GAP1*) in KC pathogenesis.

Initially, it was speculated that association with KC of rs4954218 arises from unidentified causative variants interfering with *RAB3GAP1* gene functionality, the latter being a good candidate gene based on its biological function [7]. However, according to the GRCh38 human genome assembly, this SNP is intronic to a novel protein coding gene *MAP3K19* (mitogen-activated protein kinase kinase kinase 19). Studies have shown that MAP kinases regulate neuronal differentiation by activating transcription factors such as AP-1 [18,19]. Interestingly, transcriptome analysis of KC corneas revealed that 69 of 87 genes with reduced expression compared to control tissues are regulated by AP-1 [20]. The effect of rs4954218 maybe therefore associated with pathways unrelated to *RAB3GAP1*. However, until the exact mechanisms between KC pathogenesis and the locus tagged by rs4954218 are known, this remains hypothetical.

The study is limited by the relatively modest sample size, which may be the reason for inability to replicate some of the SNPs in the Czech cases-control panel. However, the original GWAS were based on relatively small number of cases which often leads to type I or II errors. Another limitation is the usage of population controls instead of ophthalmologically examined subjects although given the estimated prevalence of KC 1:2,000 [2] it is not very likely that an individual with an unrecognized KC phenotype would be included. In conclusion, the current replication study has positively validated association of several SNPs with KC. The results of this study raise our confidence in the relevance of association of these genetic loci with KC and should pave the way to further studies that are needed to assess the functional mechanisms and direct biological relevance of the genetic variants behind that association.

## Supporting information

S1 Table Previously published association studies on SNPs investigated in the current study with keratoconus. (DOCX)

S2 Table Previously published association studies on SNPs influencing central corneal thickness investigated in the current study and their testing in keratoconus. (DOCX)

S3 Table Primer sequences used for internal data quality assessment. (DOCX)
