# Supplementary material for: Replication of SNP associations with keratoconus in a Czech cohort
Source: PLoS One. 2017 Feb 16;12(2):e0172365. doi: 10.1371/journal.pone.0172365 (PMC5313182; doi:10.1371/journal.pone.0172365)
Supplement: S1 Table — (DOCX) [file pone.0172365.s001.docx]

**S1 Table. Previously published association studies on SNPs investigated in the current study** **with keratoconus.** Only results provided for SNPs discovered by genome-wide association studies (GWAS) and subsequently tested in independent case-control panels are shown. Directions of A1 allele effects are provided for p-values <0.05.

| **SNP ID**  **Nearest gene** | **Population** | **A1/A2** | **KC cases No.** | **Controls No.** | **A1 frequency cases** | **A1 frequency controls** | **OR** | **Reported p-value** | **A1 effect** | **Genotyping method** | **Reference** |
| --- | --- | --- | --- | --- | --- | --- | --- | --- | --- | --- | --- |
| rs4839200  ***KCND3*** (31.7 kb upstream) | US white | A/G | 222 | 3,324 | 0.220 | 0.130 | 1.79 | 1.1 x 10^-6^ | Risk | **GWAS** - Illumina HumanHap370 array | [1] |
|  | Australian white |  | 524 |  | 0.165 | 0.121 | 1.43 | 1.4 x 10^-4^ | Risk | Sequenom iPlex assay | [2] |
|  |  |  |  | 2,761 |  |  |  |  |  | Illumina HumanHap610 array |  |
| rs4954218  ***RAB3GAP1***  (6.4 kb upstream)  ***MAP3K19*** (intron 1; c.-480+1330C>A) | US white | G/T | 222 | 3,324 | 0.140 | 0.250 | 0.50 | 2.4 x 10^-7^ | Protective | **GWAS** - Illumina HumanHap370 array | [1] |
|  | US white |  | 304 | 518 | 0.170 | 0.230 | 0.69 | 0.004 | Protective | Custom BeadChip Illumina iSelect Infinium |  |
|  | Australian white |  | 524 |  | 0.251 | 0.306 | 0.76 | 3.5 x 10^-4^ | Protective | Sequenom iPlex assay | [2] |
|  |  |  |  | 2,761 |  |  |  |  |  | Illumina HumanHap610 array |  |
|  | Chinese |  | 210 | 191 | 0 | 0 | - | - | - | Sequenom iPlex assay | [3] |
| rs757219  ***IMMPL2***  (intron 4; c.240-118861C>T) | US white | G/A | 222 | 3,324 | 0.230 | 0.150 | 1.70 | 6 x 10^-6^ | Risk | **GWAS** - Illumina HumanHap370 array | [1] |
|  | US white |  | 304 | 518 | 0.220 | 0.170 | 1.33 | 0.03 | Risk | Custom BeadChip Illumina iSelect Infinium |  |
|  | Australian white |  | 524 |  | 0.174 | 0.136 | 1.34 | 0.001 | Risk | Sequenom iPlex assay | [2] |
|  |  |  |  | 2,761 |  |  |  |  |  | Illumina HumanHap610 array |  |
| rs214884  ***IMMPL2*** (intron 4; c.240-142987G>A) | US white | C/T | 222 | 3,324 | 0.160 | 0.090 | 1.88 | 2.4 x 10^-6^ | Risk | **GWAS** - Illumina HumanHap370 array | [1] |
|  | US white |  | 304 | 518 | 0.130 | 0.100 | 1.21 | 0.23 | - | Custom BeadChip Illumina iSelect Infinium |  |
|  | Australian white |  | 524 |  | 0.110 | 0.078 | 1.45 | 0.001 | Risk | Sequenom iPlex assay | [2] |
|  |  |  |  | 2,761 |  |  |  |  |  | Illumina HumanHap610 array |  |
| rs1328089  ***DAOA*** (324.7 kb downstream; 13q33.3) | US white | C/T | 222 | 3,324 | 0.340 | 0.250 | 1.61 | 4.1 x 10^-6^ | Risk | **GWAS** - Illumina HumanHap370 array | [1] |
|  | Australian white |  | 524 |  | 0.297 | 0.251 | 1.26 | 0.002 | Risk | Sequenom iPlex assay | [2] |
|  |  |  |  | 2,761 |  |  |  |  |  | Illumina HumanHap610 array |  |
| rs1328083  ***DAOA*** (309.6 kb downstream; 13q33.3) | US white | G/T | 222 | 3,324 | 0.240 | 0.150 | 1.76 | 8.7 x 10^-7^ | Risk | **GWAS** - Illumina HumanHap370 array | [1] |
|  | US white |  | 304 | 518 | 0.220 | 0.210 | 1.04 | 0.78 | - | Custom BeadChip Illumina iSelect Infinium |  |
|  | Australian  white |  | 524 |  | 0.204 | 0.159 | 1.36 | 3.5 x 10^-4^ | Risk | Sequenom iPlex assay | [2] |
|  |  |  |  | 2,761 |  |  |  |  |  | Illumina HumanHap610 array |  |

**KC** - keratoconus; ***DAOA*** - D-amino acid oxidase activator; ***IMMP2L*** = IMP2 inner mitochondrial membrane peptidase-like (*S. cerevisiae*), NM_032549.3; ***KCND3*** = potassium channel, voltage gated Shal related subfamily D, member 3; ***MAP3K19*** = mitogen-activated protein kinase kinase kinase 19, NM_001018044.2; **OR** – odds ratio; ***RAB3GAP1*** = RAB3 GTPase activating protein subunit 1 (catalytic)

**References**

1. Li X, Bykhovskaya Y, Haritunians T, Siscovick D, Aldave A, Szczotka-Flynn L, et al. A genome-wide association study identifies a potential novel gene locus for keratoconus, one of the commonest causes for corneal transplantation in developed countries. Hum Mol Genet. 2012;21:421-429. doi: 10.1093/hmg/ddr460.
2. Bae HA, Mills RA, Lindsay RG, Phillips T, Coster DJ, Mitchell P, et al. Replication and meta-analysis of candidate loci identified variation at RAB3GAP1 associated with keratoconus. Invest Ophthalmol Vis Sci. 2013;54:5132-5135. doi: 10.1167/iovs.13-12377.
3. Hao XD, Chen P, Chen YL, Li SX, Wang Z. Evaluating the Association between Keratoconus and Reported Genetic Loci in a Han Chinese Population. Ophthalmic Genet. 2015 Jun;36(2):132-6. doi: 10.3109/13816810.2015.1005317.
