# Supplementary material for: Replication of SNP associations with keratoconus in a Czech cohort
Source: PLoS One. 2017 Feb 16;12(2):e0172365. doi: 10.1371/journal.pone.0172365 (PMC5313182; doi:10.1371/journal.pone.0172365)
Supplement: S2 Table — (DOCX) [file pone.0172365.s002.docx]

**S2 Table. Previously published association studies on SNPs influencing central corneal thickness investigated in the current study** **and their testing in keratoconus.** Only results provided for independent case-control panels and SNPs discovered by genome-wide association studies (GWAS) are shown. Directions of A1 allele effects are shown for p-values <0.05.

| **SNP ID**  **Nearest gene** | **Population** | **A1/A2** | **KC cases**  **No.** | **Controls**  **No.** | **A1 frequency**  **cases** | **A1 frequency**  **controls** | **OR** | **Reported**  **p-value** | **A1 effect** | **Genotyping method** | **Reference** |
| --- | --- | --- | --- | --- | --- | --- | --- | --- | --- | --- | --- |
| rs4894535  ***FNDC3B*** (intron 6; c.791-8111C>T) | US white | T/C | 222 | 3,324 | 0.210 | 0.150 | 1.52 | 8.5 x 10^-4^ | Risk | **GWAS** - Illumina HumanHap370 array | [1] |
|  | Australian white |  |  | 2,761 | 0.210 | 0.160 | 1.46 | 1.3 x 10^-6^ | Risk | Illumina HumanHap610 array |  |
|  | Australian and Irish |  | 517 + 135 |  |  |  |  |  |  | Sequenom iPlex assay |  |
|  | Chinese |  | 199 | 178 | 0.394 | 0.357 | NA | 0.143 | - | Sequenom iPlex assay | [2] |
|  | Saudi Arabian |  | 108 | 300 | 0.160 | 0.120 | 1.4 | 0.1 | - | TaqMan assay | [3] |
| rs1536482  ***COL5A1*** (93.1 kb upstream) | US white |  | 222 | 3,324 | 0.410 | 0.330 | 1.32 | 6.5 x 10^-3^ | Risk | **GWAS** - Illumina HumanHap370 array | [1] |
|  | Australian white | A/G |  | 2,761 | 0.400 | 0.340 | 1.32 | 1.2 x 10^-5^ | Risk | Illumina HumanHap610 array |  |
|  | Australian and Irish |  | 517 + 135 |  |  |  |  |  |  | Sequenom iPlex assay |  |
|  | US white |  | 304 | 518 | 0.420 | 0.360 | 1.28 | 0.02 | Risk | Custom BeadChip Illumina iSelect Infinium | [4] |
|  | Australian white |  | 157 | 673 | 0.330 | 0.290 | 1.15 | 0.387 | - | SpectroCHIP II microarray | [5] |
|  |  |  |  |  |  |  |  |  |  |  |  |
|  | Chinese |  | 207 | 191 | 0.229 | 0.215 | NA | 0.184 | - | Sequenom iPlex assay | [2] |
|  | Saudi Arabian |  | 108 | 300 | 0.450 | 0.410 | 1.1 | 0.4 | - | TaqMan assay | [3] |
| rs1324183  ***MPDZ*** (277.8 kb upstream) | US white | A/C | 222 | 3,324 | 0.260 | 0.210 | 1.33 | 0.02 | Risk | **GWAS** - Illumina HumanHap370 array | [1] |
|  | Australian white |  |  | 2,761 | 0.240 | 0.200 | 1.33 | 8.8 x 10^-5^ | Risk | Illumina HumanHap610 array |  |
|  | Australian and Irish white |  | 517 + 135 |  |  |  |  |  |  | Sequenom iPlex assay |  |
|  | Australian white |  | 157 | 673 | 0.290 | 0.190 | 1.68 | 0.001 | Risk | SpectroCHIP II microarray | [5] |
|  | Chinese |  | 208 | 190 | 0.288 | 0.211 | NA | 0.015 | Risk | Sequenom iPlex assay | [2] |
|  | Saudi Arabian |  | 108 | 300 | 0.250 | 0.280 | 0.8 | 0.4 | - | TaqMan assay | [3] |
| rs2721051  ***FOXO1*** (18.9 kb downstream) | US white | T/C | 222 | 3,324 | 0.170 | 0.100 | 1.83 | 9.5 x 10^-6^ | Risk | **GWAS** - Illumina HumanHap370 array | [1] |
|  | Australian white |  |  | 2,761 | 0.140 | 0.100 | 1.53 | 3.2 x 10^-6^ | Risk | Illumina HumanHap610 array |  |
|  | Australian and Irish white |  | 517 + 135 |  |  |  |  |  |  | Sequenom iPlex assay |  |
|  | Australian white |  | 157 | 673 | 0.150 | 0.100 | 1.45 | 0.057 | - | SpectroCHIP II microarray | [5] |
|  | Chinese |  | 206 | 188 | 0 | 0.003 | NA | 0.477 | - | Sequenom iPlex assay | [2] |
|  | Saudi Arabian |  | 108 | 300 | 0.140 | 0.110 | 1.4 | 0.2 | - | TaqMan assay | [3] |
| rs9938149  ***ZNF469*** (129.4 kb upstream) | US white | A/C | 222 | 3,324 | 0.330 | 0.270 | 1.52 | 2.5 x 10^-3^ | Risk | **GWAS** - Illumina HumanHap370 array | [1] |
|  | Australian white |  |  | 2,761 | 0.680 | 0.640 | 1.19* | 7.1 x 10^-3^* | Risk | Illumina HumanHap610 array |  |
|  | Australian and Irish white |  | 517 + 135 |  |  |  |  |  |  | Sequenom iPlex assay |  |
|  | Australian white |  | 157 | 673 | 0.720 | 0.620 | 1.47 | 0.01 | Risk | SpectroCHIP II microarray | [5] |
|  | Chinese |  | 209 | 190 | 0.930 | 0.910 | NA | 0.796 | - | Sequenom iPlex assay | [2] |
|  | Saudi Arabian |  | 108 | 300 | 0.710 | 0.680 | 0.9 | 0.4 | - | TaqMan assay | [3] |

**KC** - keratoconus; ***COL5A1*** - collagen, type V, alpha 1; ***FNDC3B*** *-* fibronectin type III domain containing 3B, NM_022763.3; ***FOXO1*** - forkhead box O1; **KC -** keratoconus; ***MPDZ*** - multiple PDZ domain protein; **NA** – not available; **OR** – odds ratio; ***ZNF469*** - zinc finger protein 469

**References**

1. Lu Y, Vitart V, Burdon KP, Khor CC, Bykhovskaya Y, Mirshahi A, et al. Genome-wide association analyses identify multiple loci associated with central corneal thickness and keratoconus. Nat Genet. 2013;45:155-163. doi: 10.1038/ng.2506.
2. Hao XD, Chen P, Chen YL, Li SX, Wang Z. Evaluating the Association between Keratoconus and Reported Genetic Loci in a Han Chinese Population. Ophthalmic Genet. 2015 Jun;36(2):132-6. doi: 10.3109/13816810.2015.1005317.
3. Abu-Amero KK, Helwa I, Al-Muammar A, Strickland S, Hauser MA, Allingham RR, et al. Case-control association between CCT-associated variants and keratoconus in a Saudi Arabian population. J Negat Results Biomed. 2015 Jun 4;14:10. doi: 10.1186/s12952-015-0029-5.
4. Li X, Bykhovskaya Y, Canedo AL, Haritunians T, SiscovickD, Aldace AJ, et al. Genetic association of COL5A1 variants in keratoconus patients suggests a complex connection between corneal thinning and keratoconus. Invest Ophthalmol Vis Sci. 2013;54:2696-2704. doi: 10.1167/iovs.13-11601.
5. Sahebjada S, Schache M, Richardson AJ, Snibson G, MacGregor S, Daniell M, et al. Evaluating the association between keratoconus and the corneal thickness genes in an independent Australian population. Invest Ophthalmol Vis Sci. 2013;54:8224-8228. doi: 10.1167/iovs.13-12982.
