# Supplementary material for: Replication of SNP associations with keratoconus in a Czech cohort
Source: PLoS One. 2017 Feb 16;12(2):e0172365. doi: 10.1371/journal.pone.0172365 (PMC5313182; doi:10.1371/journal.pone.0172365)
Supplement: S3 Table — (DOCX) [file pone.0172365.s003.docx]

**S3 Table. Primer sequences used for internal data quality assessment.**

|  | **FWD sequence 5’-3’** | **REV sequence 5’-3’** | **Product size** |
| --- | --- | --- | --- |
| **rs1328083** | TGGTGTCCTTTCTCACCACA | GAAAATGCATTGGTGTTTCATC | 196 |
| **rs1328089** | TCCCAGTCACCTTGACAAAA | GCTGTGGGTTGGTGCTTTAT | 232 |
| **rs1536482** | AGGTCCCTTGAGCCCTTTTA | TGGAGGGTGAGTGCTATGGT | 173 |
| **rs4894535** | TGTCTGCCTTACACCCATGA | TACCACCCCAAGAAATCAGC | 221 |
| **rs2721051** | AAGGGAAGAGGCAAATGTGA | CCAAGGTTAACCGAAGTCCA | 250 |
| **rs214884** | TGCAAGATCTGTAAGGGCAGT | ATCACCATTTTTGCCATGTG | 235 |
| **rs757219** | GCTCAAACCATGAGGATTGC | ATAACTTGGAATGCGGCAAA | 153 |
| **rs4839200** | GCCAGCGTCAGGTTTTTACT | TGGCCCTCAAATCTTCTCTG | 245 |
| **rs4954218** | TGTGGTCATTATTTGGCCATT | TTTGAGGCTGCAGTGAGCTA | 225 |
| **rs9938149** | GGTGACAACTGCCATCCTTT | CGGGGTTACATCTGCAATTC | 196 |
| **rs1324183** | TATTGATCCACAGCCAGCAG | AAGCGCTTCTAAAAGCCAATC | 235 |
